# ClinGen Pathogenicity Calculator: a configurable system for assessing pathogenicity of genetic variants

**DOI:** 10.1186/s13073-016-0391-z

**Published:** 2017-01-12

**Authors:** Ronak Y. Patel, Neethu Shah, Andrew R. Jackson, Rajarshi Ghosh, Piotr Pawliczek, Sameer Paithankar, Aaron Baker, Kevin Riehle, Hailin Chen, Sofia Milosavljevic, Chris Bizon, Shawn Rynearson, Tristan Nelson, Gail P. Jarvik, Heidi L. Rehm, Steven M. Harrison, Danielle Azzariti, Bradford Powell, Larry Babb, Sharon E. Plon, Aleksandar Milosavljevic, on behalf of the ClinGen Resource

**Affiliations:** 1 Baylor College of Medicine, Houston, TX 77030 USA; 2 The Renaissance Computing Institute, The University of North Carolina at Chapel Hill, Chapel Hill, NC 27517 USA; 3 University of Utah Hospitals and Clinics, University of Utah, Salt Lake City, UT 84112 USA; 4 Geisinger autism and developmental medicine, Lewisburg, PA 17837 USA; 5 Laboratory for Molecular Medicine, Partners HealthCare Personalized Medicine, Cambridge, MA 02139 USA; 6 Brigham & Women’s Hospital and Harvard Medical School, Boston, MA 02115 USA; 7 Department of Genetics, The University of North Carolina at Chapel Hill, Chapel Hill, NC 27514 USA; 8 GeneInsight, Sunquest Information System, Boston, MA 02210 USA; 9 Division of Medical Genetics, Department of Medicine, University of Washington, Seattle, WA 98195 USA

**Keywords:** Genome sequencing, Exome sequencing, Clinical genome sequencing, Clinical exome sequencing, ACMG guidelines, ClinVar, ClinGen, Clinical Genome Resource, Knowledge commons, Data commons, Data sharing, Linked Data, Big Data

## Abstract

**Background:**

The success of the clinical use of sequencing based tests (from single gene to genomes) depends on the accuracy and consistency of variant interpretation. Aiming to improve the interpretation process through practice guidelines, the American College of Medical Genetics and Genomics (ACMG) and the Association for Molecular Pathology (AMP) have published standards and guidelines for the interpretation of sequence variants. However, manual application of the guidelines is tedious and prone to human error. Web-based tools and software systems may not only address this problem but also document reasoning and supporting evidence, thus enabling transparency of evidence-based reasoning and resolution of discordant interpretations.

**Results:**

In this report, we describe the design, implementation, and initial testing of the Clinical Genome Resource (ClinGen) Pathogenicity Calculator, a configurable system and web service for the assessment of pathogenicity of Mendelian germline sequence variants. The system allows users to enter the applicable ACMG/AMP-style evidence tags for a specific allele with links to supporting data for each tag and generate guideline-based pathogenicity assessment for the allele. Through automation and comprehensive documentation of evidence codes, the system facilitates more accurate application of the ACMG/AMP guidelines, improves standardization in variant classification, and facilitates collaborative resolution of discordances. The rules of reasoning are configurable with gene-specific or disease-specific guideline variations (e.g. cardiomyopathy-specific frequency thresholds and functional assays). The software is modular, equipped with robust application program interfaces (APIs), and available under a free open source license and as a cloud-hosted web service, thus facilitating both stand-alone use and integration with existing variant curation and interpretation systems. The Pathogenicity Calculator is accessible at http://calculator.clinicalgenome.org.

**Conclusions:**

By enabling evidence-based reasoning about the pathogenicity of genetic variants and by documenting supporting evidence, the Calculator contributes toward the creation of a knowledge commons and more accurate interpretation of sequence variants in research and clinical care.

**Electronic supplementary material:**

The online version of this article (doi:10.1186/s13073-016-0391-z) contains supplementary material, which is available to authorized users.

## Background

Successful clinical diagnostic application of exome and genome sequencing hinges on the ability of clinical laboratories to consistently and accurately assess pathogenicity of genetic variants. Several studies have identified inconsistencies in variant classification across laboratories, reference databases, and even within individual databases [1–4]. Interpretation requires the use of professional judgment and non-standardized evidence types, thus it is unrealistic to expect interpretation concordance of all variants across all laboratories and data sources. However, it is anticipated that knowledge sharing, standardization of best practices, and transparent documentation of evidence-based reasoning will have significant positive impact on both consistency and accuracy.

To improve the standardization of variant interpretation by clinical testing laboratories, the American College of Medical Genetics (ACMG) and the Association of Molecular Pathologists (AMP) have recently updated guidelines for the interpretation of sequence variants [5]. The new guidelines provide significantly more information about the types and weight of evidence used to classify variants compared to the prior guidelines [6]. Overall, they define 28 different evidence codes capturing different types of evidence for or against pathogenicity of a variant, only a subset of codes typically being applicable for a specific variant. There are 20 rules for combining the evidence codes for a given variant in order to reach one of five conclusions about pathogenicity (Pathogenic, Likely Pathogenic, Uncertain Significance, Likely Benign, or Benign).

The formal structuring of evidence-based arguments for or against pathogenicity and their implementation in software provide several opportunities for enhancing the emerging knowledge of genetic variation. One such opportunity is efficient evidence tracking that facilitates critical evaluation of variant classifications. Recent studies [1, 4] suggests that a framework for organizing and communicating evidential support and the reasoning behind specific assessments of variant pathogenicity facilitates resolution of inter-laboratory discordances. However, currently available software systems and web services provide only rudimentary support for guideline-based reasoning [7] and do not facilitate full evidence tracking or collaborative resolution of discordances. They also do not enable guideline configuration to meet ACMG guideline specification and customization for specific genes and diseases, nor integration with existing variant curation systems. We here address these gaps by describing the design, development, and application of the ClinGen Pathogenicity Calculator, thus fostering the improvement of clinical classification of variants, a key goal of the NIH supported Clinical Genome Resource (ClinGen) [8].

## Implementation

### The main interface

**Fig. 1:**
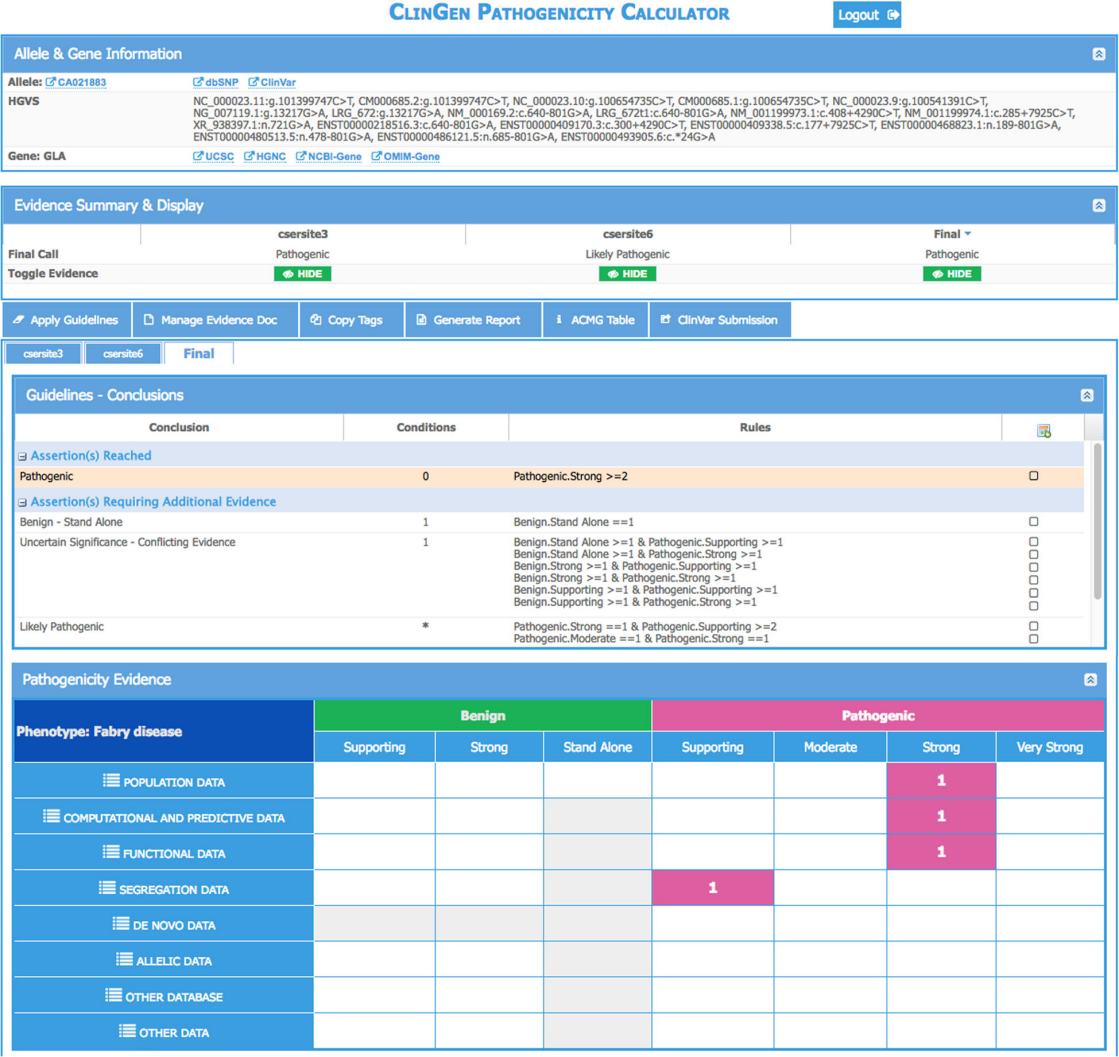
Four-panel interface for the ClinGen Pathogenicity Calculator

The Calculator client-side, including the main interface, is implemented using JavaScript and ExtJS. The main Calculator interface (Fig. 1) presents variant assessment information within four vertically stacked panels. The top panel displays information available in the ClinGen Allele Registry for the variant being classified plus links to additional information about the variant in key external resources. The second panel provides an assessment summary of the current user plus any other assessment summaries for the same variant that are shared within collaborating groups or publicly available. In this panel, one may either initiate a new assessment by creating a new blank evidence document (assessment record) or browse available assessments by clicking on the appropriate tab. The third panel provides a summary of ACMG/AMP guideline rules that are satisfied to draw a classification based on the evidence tags that were provided for this variant in the evidence document, with each evidence tag corresponding to an evidence code. The last panel provides a summary of evidence tags that were provided, each displayed within one cell of the evidence grid. The cell for each tag is determined by the strength of tag evidence for or against pathogenicity (grid columns) and by tag evidence type (grid rows). The Calculator also provides alternate classifications that could be possibly satisfied in the middle panel and in the lower panel the evidence code(s) that would be needed to reach this classification. This latter feature may aid groups doing variant classification to identify what type of additional data could be sought (or shared) in order to reach a conclusion (as illustrated in Additional file 1: Figure S1).

### Allele registration and data linking

As illustrated in Additional file 2: Figure S2, the Calculator utilizes the services of the ClinGen Allele Registry to identify a unique variant and collates information about it from external sources to aid in classification. The registry accepts wide variety of Human Genome Variation Society (HGVS) recommended expressions for substitution, insertion, deletion, and indels [9]. It also supports query using HUGO Gene Nomenclature Committee (HGNC) gene identifiers. If an allele is not present in the Registry, the Calculator may register the new allele within seconds upon request by an authenticated user. More information about the ClinGen Allele Registry is available at http://reg.clinicalgenome.org (manuscript in preparation).

The registry enables linking and viewing of pathogenicity assessments that are independently generated by different users (displayed under different tabs in the second panel in Fig. 1) as well as linking of relevant external supporting information about the variant. The current set of externally linked information includes ClinVar [10], dbSNP [11], dbNSFP [12], COSMIC [13], ExAC [14], and myvariant.info [15].

### Modeling and storage of semi-quantitative guidelines and assessments

For the purpose of modeling and storing configurable semi-quantitative guidelines and assessments for individual variants, the Calculator utilizes GenboreeKB, a system for document-oriented modeling and storage. The information is stored within four document collections within GenboreeKB. The first two collections store semi-quantitative guidelines such as the ACMG/AMP guidelines using one document collection for guideline rules and metarules and another for evidence codes. The third document collection defines the cell (row and column) for each evidence tag within the evidence grid (fourth panel in Fig. 1 or alternatively Fig. 1d). The fourth document collection stores the complete pathogenicity assessment information for individual variants.

### The reasoning engine

One core module of the Pathogenicity Calculator is a generic reasoner written in the R programming language. The reasoner takes the guidelines (defined using rules, metarules, and evidence tags) and aggregated evidence for the variant being pathogenic or benign (encoded as a set of evidence tags) and produces a conclusion (Fig. 1). The reasoner provides documentation of reasoning for each conclusion and identifies conclusions that are a few evidence tags away from being satisfied. The output of the reasoner is a JSON document that is used by the Pathogenicity Calculator interface to display the conclusion, the evidence codes, and any other conclusions that are close to being reached.

### Support for collaboration

GenboreeKB is implemented in Ruby as an enhancement to the Genboree server. Thus, the group-based data sharing is managed by the Genboree access control system. A web-based GenboreeKB management interface employing the ExtJS Javascript framework is available as a plugin of the Redmine Rails (www.redmine.org) application.

### Data modeling, storage, and application program interfaces

GenboreeKB utilizes MongoDB for data storage and exposes in an access-controlled way. The complete data contents of a Calculator is exposed ﻿as JSON via Genboree REST HTTP application program interfaces (APIs). GenboreeKB stores both data models and the data itself as document collections and exposes them via the APIs.

### Integration with existing variant curation and evaluation tools and systems

Numerous tools and systems for variant curation and evaluation have been developed and used by various laboratories. Recognizing that some of those tools may benefit from the functionalities of the Calculator, all the data and functionality of the Calculator are programmatically accessible via REST APIs. The Calculator may be used as a web service (with authentication and collaborative features such as group-based access control) or by installing it on local hardware.

## Results

### The original ACMG/AMP guidelines can be translated into an algorithm with only modest adaptations

Two adaptations of the original guidelines had to be introduced to facilitate formal algorithmic reasoning. First, several metarules were introduced to capture implicit “common sense” rules practitioners were expected to apply without explicit instruction. For example, if there are two strong and three moderate-strength evidence tags in favor of pathogenicity, then the variant can be pathogenic (≥2 strong evidence tag) or likely pathogenic (≥3 moderate-strength evidence tags). In such a case the metarules specify that the variant is pathogenic. Conversely, evidence tags that can be combined to yield both Likely Benign and Benign are scored as Benign. The Calculator distinguishes between uncertain significance that arises due to insufficient evidence from uncertain significance due to conflicting evidence.

Second, the guidelines allow the practitioners to change the strength level originally assigned to certain tags based on perceived strength of supporting data for the tag (“To provide critical flexibility to variant classification, some criteria listed as one weight can be moved to another weight using professional judgment, depending on the evidence collected,” from [5]). To accommodate this flexibility within the formal algorithmic reasoning system of the Calculator, multiple new tags were defined for each original tag where the new tags are identical except that their evidence strength was formally redefined. For example, the original “PVS1” tag constitutes “Very Strong” evidence for pathogenicity while the newly defined “PVS1-Strong” tag is defined as the same type of evidence except that it constitutes only “Strong” evidence for pathogenicity.

### The calculator reduces human error when applying variant assessment guidelines

The ACMG/AMP guidelines and the Calculator were tested in the context of a multi-site study [1] involving nine molecular diagnostic laboratories involved in the Clinical Sequencing Exploratory Research (CSER) consortium and 99 variants that were evaluated by multiple laboratories who applied the ACMG/AMP rules. The Calculator helped identify human error when these groups applied the guidelines. The Calculator identified that for 16 of 353 (5%) of the variant assessments, the ACMG/AMP evidence codes listed by the participating laboratory did not support the conclusion provided by the laboratory; of these, nine (2.5%) of the 353 were errors. The remaining seven were cases when the labs used professional judgment to override the ACMG/AMP rules.

**Fig. 2:**
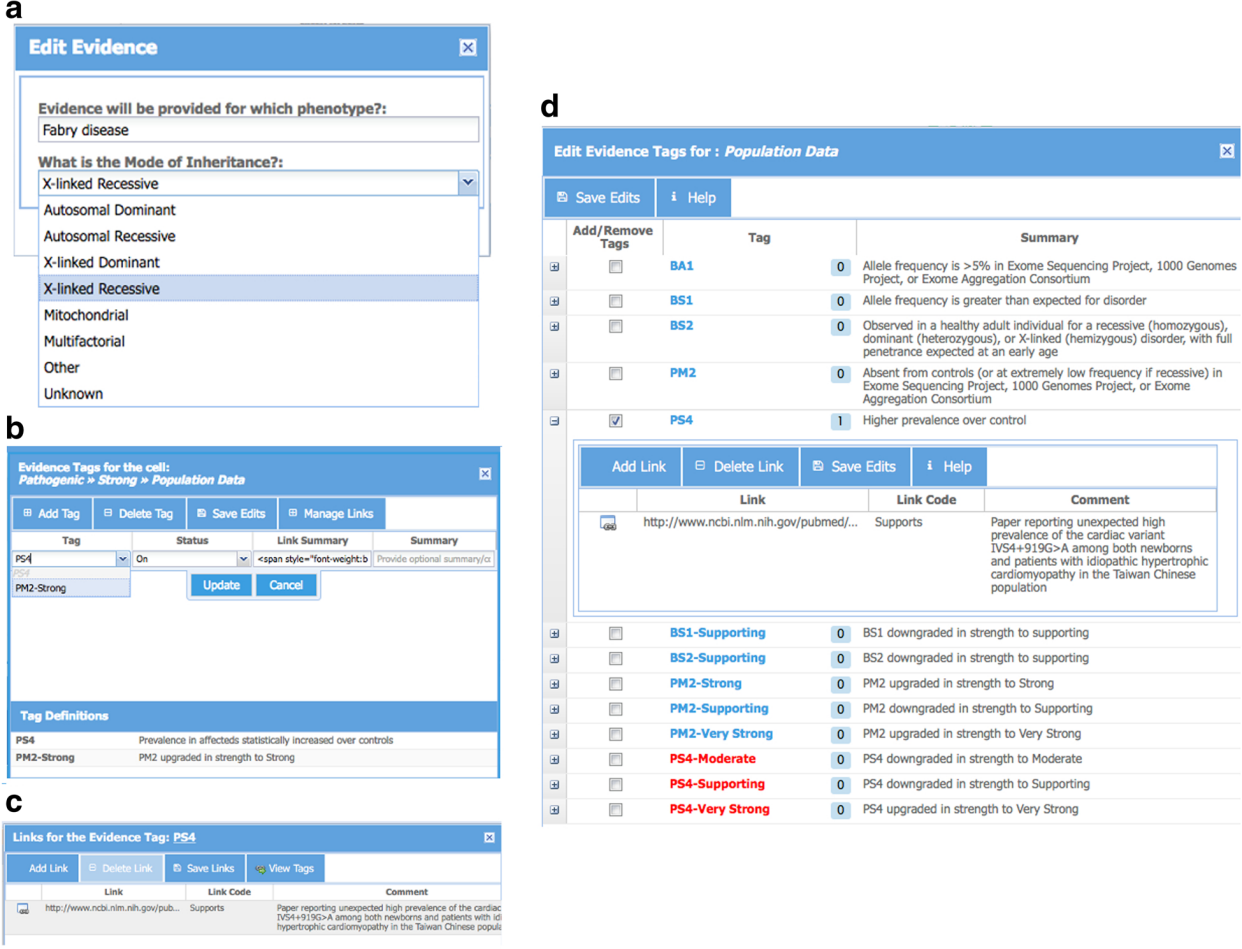
**a** Interface for initializing an evidence document for a selected allele by adding information about disease condition and mode of inheritance. **b** Interface for updating tags by clicking on a cell and selecting a tag from a pull-down menu. A link to a table defining tags in each cell (such as a table with ACMG tags) is provided for reference. **c** Interface for updating links to supporting data for a tag by opening the “Manage Links” tab in panel B and by entering one or more URLs/Links along with any free-text comments. **d** An alternate interface to manage tags and provide summary and links. This interface lists all the tags for each evidence type (e.g. Population Data, Computational Data, etc.). This panel is activated by clicking on the evidence type in the *left-most column* of the evidence summary table (Fig. 1, *left-most column* in the *bottom panel*)

A user may initiate a session by selecting a previously created assessment from a list or by creating a new assessment (user interfaces not shown). A new assessment is initiated by providing an HGVS allele identifier (for example, NM_000169.2:c.639 + 919G > A in the alpha galactosidase gene, *GLA*) and condition name and mode of inheritance (Fig. 2a). Using the services of the ClinGen Allele Registry, the Calculator identifies the allele by a canonical identifier (“CA021883” in the top panel) and, if the allele is not present in the registry, provides an option to register the new allele within seconds. Upon registration, all future assessments of the same allele become linked via the canonical identifier, thus enabling the sharing of assessments among users and between the users and any automated assessment pipelines. Shared assessments appear under different tabs in the second panel (Fig. 1) where one may also initiate a new assessment by creating a new blank evidence document (assessment record).

The user can then add evidence tags using one of two available interfaces. A click on an individual cell in Panel 4 activates a new panel where one or more tags that belong to that cell may be applied (Fig. 2b). A user may enter an optional free-text summary and may also associate evidence tags with multiple links to supporting data (Fig. 2c). Figure 2d shows a snapshot of a panel to manage tags using an alternative interface. This panel lists all possible tags for a selected type of variant interpretation data (e.g. population, functional, etc.). This interface is useful when the user does not know the exact location of a tag within the evidence grid (fourth panel in Fig. 1). Immediately upon addition or deletion of a tag (using any of the two interfaces), conclusions in the third panel (Fig. 1) are updated. The conclusions reached based on current tag selection appear highlighted at the top of this panel. A click on the icon to the right of this conclusion highlights the columns in the evidence grid (Additional file 1: Figure S1) that contain supporting evidence for the conclusion. In addition, potential conclusions that are close to being reached are listed, together with the total number of tags that need to be turned on for a conclusion to be reached. A click on the icon to the right of such conclusions highlights the columns in the evidence grid where the missing evidence tags reside (Additional file 1: Figure S1).

Advanced users (application developers) can access the functionalities of the Calculator via Genboree REST-APIs. Using the services, users can upload evidence tags for several variants at a time. The uploaded data become accessible through the interface as well as through REST-APIs. In the future, the bulk upload functionality will be made accessible even for regular users without any background in computer programming.

### The calculator facilitates critical evaluation of pathogenicity assertions

**Fig. 3:**
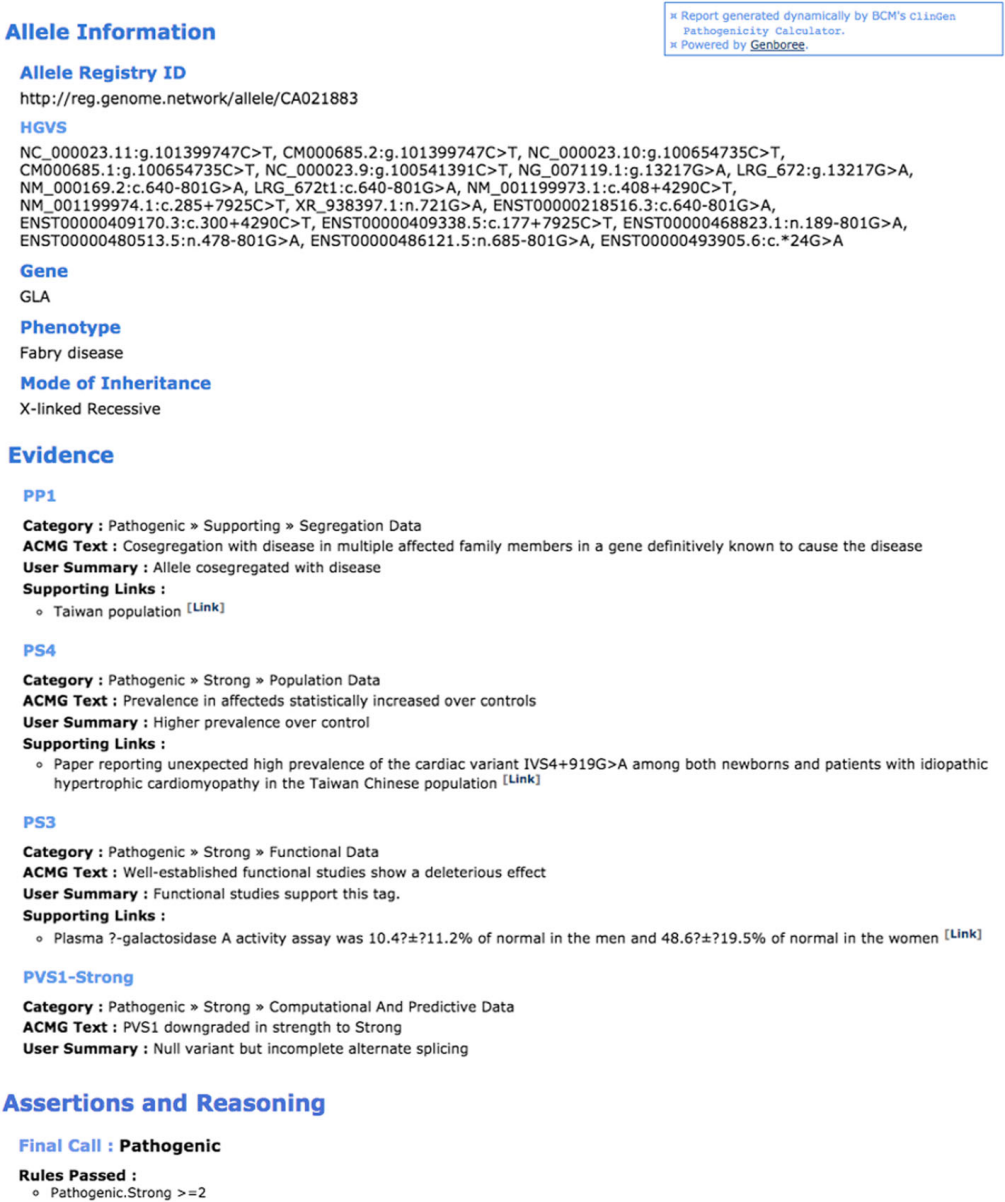
A sample summary report generated by Pathogenicity Calculator. The report itself is printable as PDF and downloadable by the user

Sharing of pathogenicity assessments of pathogenicity of variants by clinical laboratories via databases such as ClinVar aids in the practice of clinical genome interpretation. However, currently, the format of information by which pathogenicity for a given variant was determined by multiple submitters to ClinVar often appears in a free text unstructured format, if it is provided at all. It would be useful if this knowledge sharing was further extended to include the reasoning and evidence codes that supported conclusions about pathogenicity in a standardized format. For example, evidential support for an early assessment that a variant is pathogenic may not have addressed more recent information about the high frequency of the variant in a specific population, thus explaining discordance with a newer independent assessment that includes this recent information. With the evidential support available for inspection, a user of the database may benefit from being able to rapidly and critically evaluate both assessments based on the evidence provided in a structured way. Toward this goal, the Calculator facilitates the sharing of the reasoning and the evidential support behind every assessment. Access to this information is provided via the interfaces (Fig. 1, Panel 3) and also via a single comprehensive report that is also accessible via a URL and is printable as a PDF (Fig. 3). The report (as PDF) may be included in submissions to databases such as ClinVar or with Electronic Health Record (EHR) systems, thus enabling critical evaluation of conclusions about pathogenicity. Although the tool and underlying services are built in anticipation of integration with EHR and other clinical information systems by trained personnel, projects of this type have not yet been initiated.

### The calculator helps detect and resolve discordant conclusions

Recent studies indicate that the initial application of the ACMG/AMP guidelines did not increase concordance in laboratory assessment [1]; however, it did help facilitate resolution of the inter-laboratory discordances by providing a framework for organizing and communicating evidential support behind independent assessments of the same variant. To facilitate this process, the ability of the Calculator to track the reasoning and evidence discussed above is necessary but not sufficient. Two more features are essential. First, independent assessments of the same allele must be standardized, as different labs/groups may be using different transcripts or genome builds. This feature is accomplished through the services of ClinGen Allele registry (top panel, Fig. 1). Second, public or group-based sharing of assessments is critical, which is accomplished by using group access management features of the Genboree system. As a result, for each assessment for a given allele that is either owned by a user or available to him/her for public or group-based sharing, the user sees separate tabs in the second panel (Fig. 1) of the Calculator. By clicking on the tabs, the user may access complete information about other assessments (in panels 3 and 4, Fig. 1, Additional file 1: Figure S1 and Additional file 3: Figure S3). This group-based comparison of variant interpretation can also be used for proficiency testing challenges where different sites can be asked to interpret the same variant. The interpretation at the end of a challenge can then be compared to evaluate the degree of overall concordance and identify specific discordances.

The Calculator does not share information about pathogenicity of variant publicly without explicit user request. The information entered is protected using a login and password. It provides optional free-text fields summarizing tag assignments and links to supporting evidence. The Calculator does not require or have fields for providing identifying information about patients. Public sharing per user request will be enabled in future releases.

### The calculator guidelines are configurable without programming to accommodate guideline evolution and customizations for specific conditions

Although ACMG/AMP guidelines provide a general framework for Mendelian sequence variant interpretation, it was always recognized that this framework would evolve and be customized to meet requirements for specific genes and disease conditions [2, 16–19]. To address these anticipated needs, the Calculator is designed to support guideline evolution and customization without programming. By using a lower-level GenboreeKB interface (not shown), an “admin” user may fully configure the Calculator by editing the following three structured documents: (1) guideline rules and metarules; (2) evidence codes and their assignments to the columns and rows of the evidence grid (fourth panel in Fig. 1); and (3) the rows and columns of the evidence grid itself. These three documents completely define a formal “guideline” used by the Calculator. Considering this configurability, it is possible to customize the Calculator for specific purposes (e.g. to implement guidelines for somatic variant interpretation and/or multifactorial inheritance).

While each assessment refers to a specific guideline, different guidelines may exist within the same system. For example, for the purpose of assessing variants within genes causing inborn errors of metabolism, metabolite levels could be used as an evidence type in pathogenicity assessments. Because the original ACMG/AMP guidelines do not specify this type of evidence or potentially others that will be added in the future, we have designed flexibility into the Calculator to allow for addition of new evidence types (new rows in the evidence grid) and corresponding evidence tags and of new rules without programming.

## Discussion

By automating the formal guideline-based assessment of genetic variants, the ClinGen Pathogenicity Calculator can reduce human error. The Calculator extends functionality of other currently available calculators [7] along several dimensions. First, it enables critical evaluation of pathogenicity assessments by documenting the complete reasoning from conclusions to relevant rules, evidence tags and supporting data needed to reach a more definitive conclusion (e.g. from likely pathogenic to pathogenic; Additional file 1: Figure S1). Second, the Calculator facilitates collaborative resolution of discordances by linking multiple independent assessments of the same variant and by enabling their group-based sharing. Third, by being configurable, the Calculator accommodates evolution of ACMG/AMP guidelines and their customizations for specific genes and diseases [1, 16–18]. Fourth, the modular reasoning engine facilitates integration of more quantitative guidelines such as those based on Bayesian inference that are used by the community for the assessment of some cancer predisposition genes [20]. Finally, the Calculator supports programmatic integration of rule-based reasoning into existing variant interpretation and curation systems by providing robust APIs implemented using free open source software. Efforts are under way to integrate one or more functionalities of the Calculator within the variant curation interface that is under development by the ClinGen Resource.

## Conclusions

By providing an intuitive user interface and by addressing other key computational requirements, the Calculator facilitates formal and collaborative evidence-based reasoning about the pathogenicity of genetic variants, thus paving the way toward more effective and accurate clinical genomic interpretation.

## Additional files

Additional file 1: Figure S1. A *schematic* showing a visual of evidence, assertion, and reasoning for the user csersite6. The evidence, assertion, and reasoning given in this panel is visible when the tab is switched from Final (see Fig. 1) to csersite6. The “Supporting” and “Very Strong” *columns* are highlighted after clicking on the top icon on the right of the “Pathogenic” rule, indicating the type of evidence still required for the rule to be satisfied and for the variant to be classified as “Pathogenic.” (JPG 685 kb)
Additional file 2: Figure S2. Implementation of the ClinGen Pathogenicity Calculator. **A** An *abstract diagram* showing inputs and outputs. **B** Components of the Calculator, associated database, and web services. The direction of *arrows* shows data flow and component interactions. (JPG 2630 kb)
Additional file 3: Figure S3. A *schematic* showing a visual of evidence, assertion, and reasoning for the user csersite3. The evidence, assertion, and reasoning given in this panel is visible when the tab is switched from Final (as in Fig. 1) to csersite3. (JPG 691 kb)
