# Supplementary figures and images for: ClinGen Pathogenicity Calculator: a configurable system for assessing pathogenicity of genetic variants
Source: Genome Med. 2017 Jan 12;9:3. doi: 10.1186/s13073-016-0391-z (PMC5228115; doi:10.1186/s13073-016-0391-z)

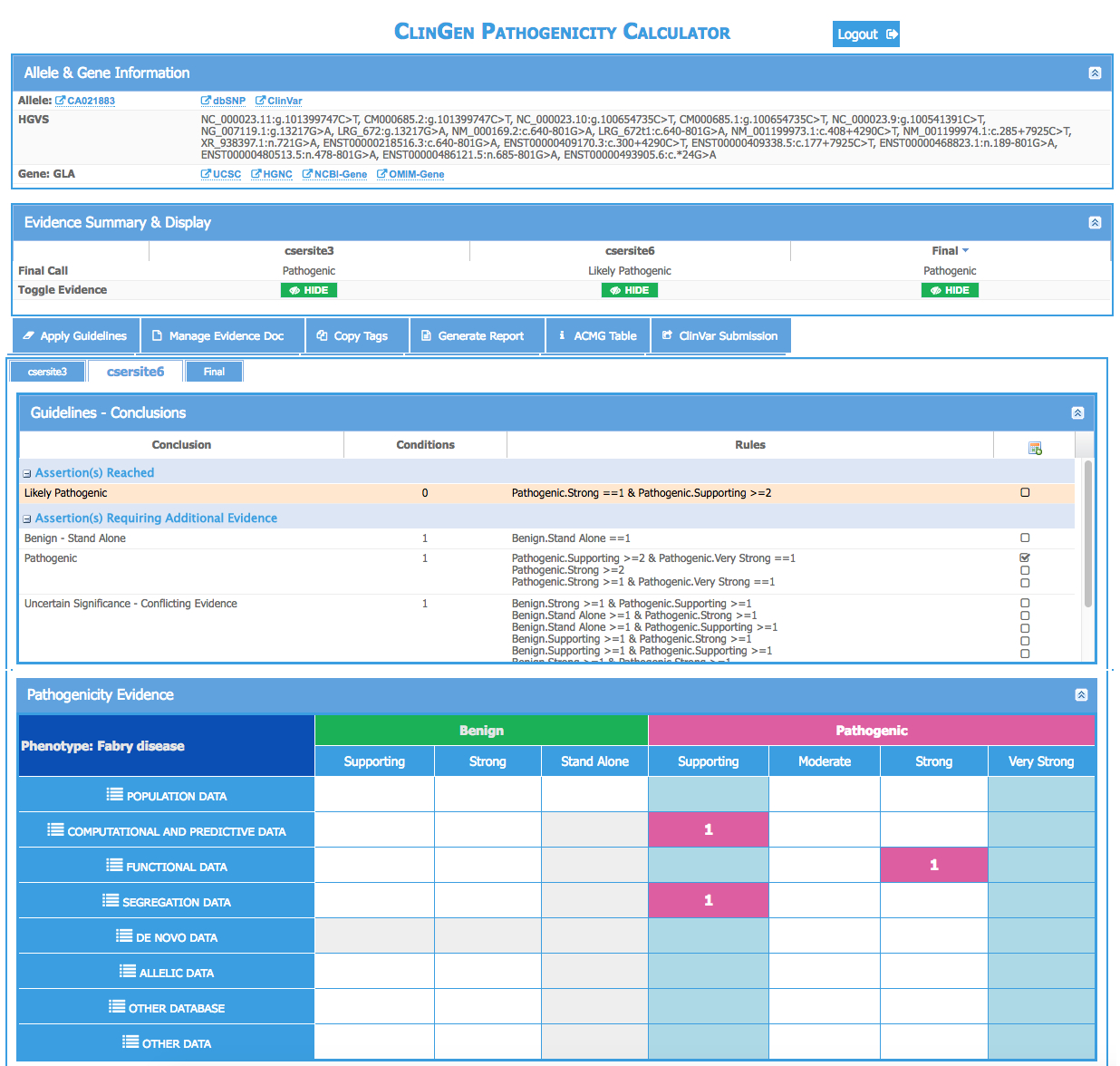

Supplement: Additional file 1: Figure S1. — A schematic showing a visual of evidence, assertion, and reasoning for the user csersite6. The evidence, assertion, and reasoning given in this panel is visible when the tab is switched from Final (see Fig. 1) to csersite6. The “Supporting” and “Very Strong” columns are highlighted after clicking on the top icon on the right of the “Pathogenic” rule, indicating the type of evidence still required for the rule to be satisfied and for the variant to be classified as “Pathogenic.” (JPG 685 kb) [file 13073_2016_391_MOESM1_ESM.jpg]

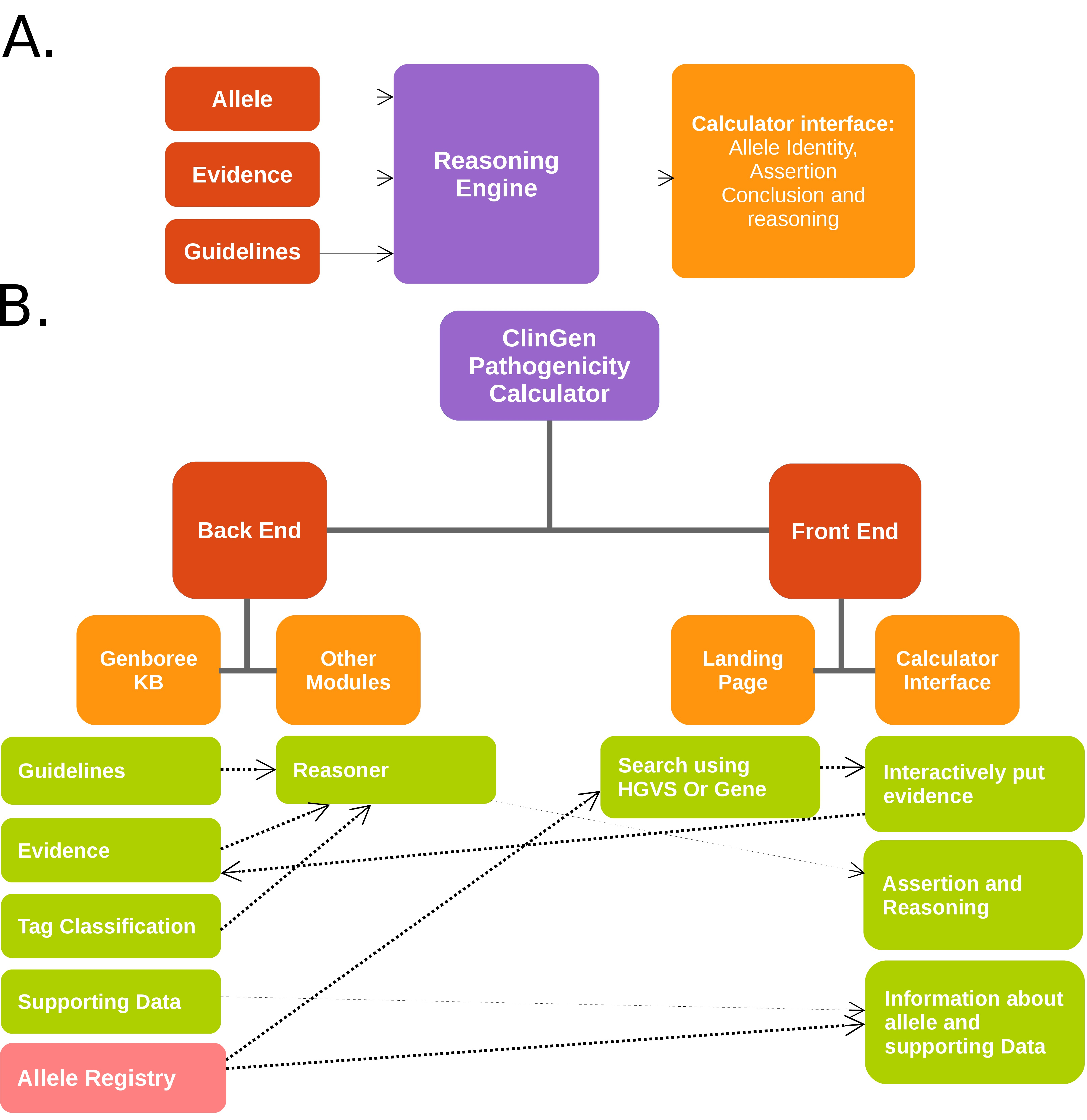

Supplement: Additional file 2: Figure S2. — Implementation of the ClinGen Pathogenicity Calculator. A An abstract diagram showing inputs and outputs. B Components of the Calculator, associated database, and web services. The direction of arrows shows data flow and component interactions. (JPG 2630 kb) [file 13073_2016_391_MOESM2_ESM.jpg]

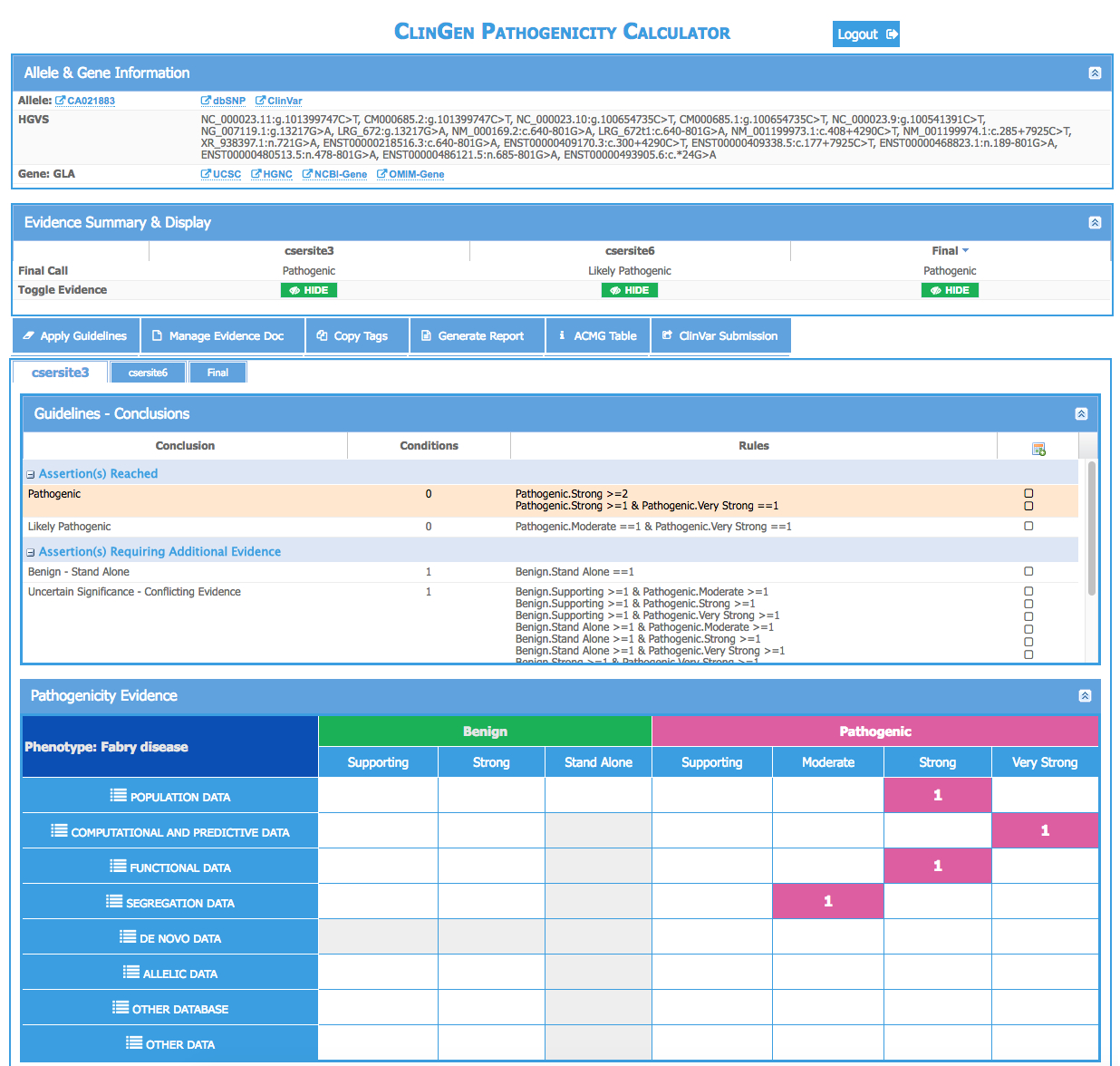

Supplement: Additional file 3: Figure S3. — A schematic showing a visual of evidence, assertion, and reasoning for the user csersite3. The evidence, assertion, and reasoning given in this panel is visible when the tab is switched from Final (as in Fig. 1) to csersite3. (JPG 691 kb) [file 13073_2016_391_MOESM3_ESM.jpg]
